# Immunity against sexual stage *Plasmodium falciparum* and *Plasmodium vivax* parasites

**DOI:** 10.1111/imr.12828

**Published:** 2019-12-16

**Authors:** Roos M. de Jong, Surafel K. Tebeje, Lisette Meerstein‐Kessel, Fitsum G. Tadesse, Matthijs M. Jore, Will Stone, Teun Bousema

**Affiliations:** ^1^ Radboud Institute for Molecular Life Sciences Radboud University Medical Center Nijmegen The Netherlands; ^2^ Armauer Hansen Research Institute Addis Ababa Ethiopia; ^3^ Radboud Institute for Health Sciences Radboud University Medical Center Nijmegen The Netherlands; ^4^ Centre for Molecular and Biomolecular Informatics Radboud Institute for Molecular Life Sciences Nijmegen The Netherlands; ^5^ Department of Immunology and Infection London School of Hygiene and Tropical Medicine London UK

**Keywords:** gametocytes, immunity, *Plasmodium falciparum*, *Plasmodium vivax*, transmission, vaccines

## Abstract

The efficient spread of malaria from infected humans to mosquitoes is a major challenge for malaria elimination initiatives. Gametocytes are the only *Plasmodium* life stage infectious to mosquitoes. Here, we summarize evidence for naturally acquired anti‐gametocyte immunity and the current state of transmission blocking vaccines (TBV). Although gametocytes are intra‐erythrocytic when present in infected humans, developing *Plasmodium falciparum* gametocytes may express proteins on the surface of red blood cells that elicit immune responses in naturally exposed individuals. This immune response may reduce the burden of circulating gametocytes. For both *P. falciparum* and *Plasmodium vivax*, there is a solid evidence that antibodies against antigens present on the gametocyte surface, when co‐ingested with gametocytes, can influence transmission to mosquitoes. Transmission reducing immunity, reducing the burden of infection in mosquitoes, is a well‐acknowledged but poorly quantified phenomenon that forms the basis for the development of TBV. Transmission enhancing immunity, increasing the likelihood or intensity of transmission to mosquitoes, is more speculative in nature but is convincingly demonstrated for *P. vivax*. With the increased interest in malaria elimination, TBV and monoclonal antibodies have moved to the center stage of malaria vaccine development. Methodologies to prioritize and evaluate products are urgently needed.

## 1 INTRODUCTION

Malaria is one of the few infectious diseases earmarked for worldwide eradication by the World Health Organization (WHO).^1^, ^2^ The majority of the malaria cases are caused by infection with *Plasmodium falciparum* or *Plasmodium vivax.* While *P. falciparum* is the dominant *Plasmodium* species in most of Africa and is associated with the most severe morbidity and mortality, *P. vivax* is more widely distributed and is increasingly recognized as an important source of morbidity and restrained economic productivity.^3^ Malaria control efforts in the recent decades, including improved access to efficacious treatment and vector control, were followed by significant reductions in malaria burden^4^ and stimulated malaria elimination initiatives. Despite these successes, the WHO estimates that there were 219 million new malaria cases and 435 000 malaria‐related deaths in 2017.^5^ This figure has remained fairly stable since 2015 indicating that progress has plateaued; some countries even experience recent increases in malaria burden and several more are off track in their elimination efforts.^5^ The emergence of parasite resistance to antimalarials^6^, ^7^ and mosquito resistance to insecticides^8^ are important threats to recent gains. One of the major challenges for malaria elimination initiatives is the very efficient spread of malaria from infected humans to mosquitoes.^1^ Interventions that target this process and interrupt transmission to mosquitoes may be crucial to achieve elimination in many areas.^9^

**Figure 1:**
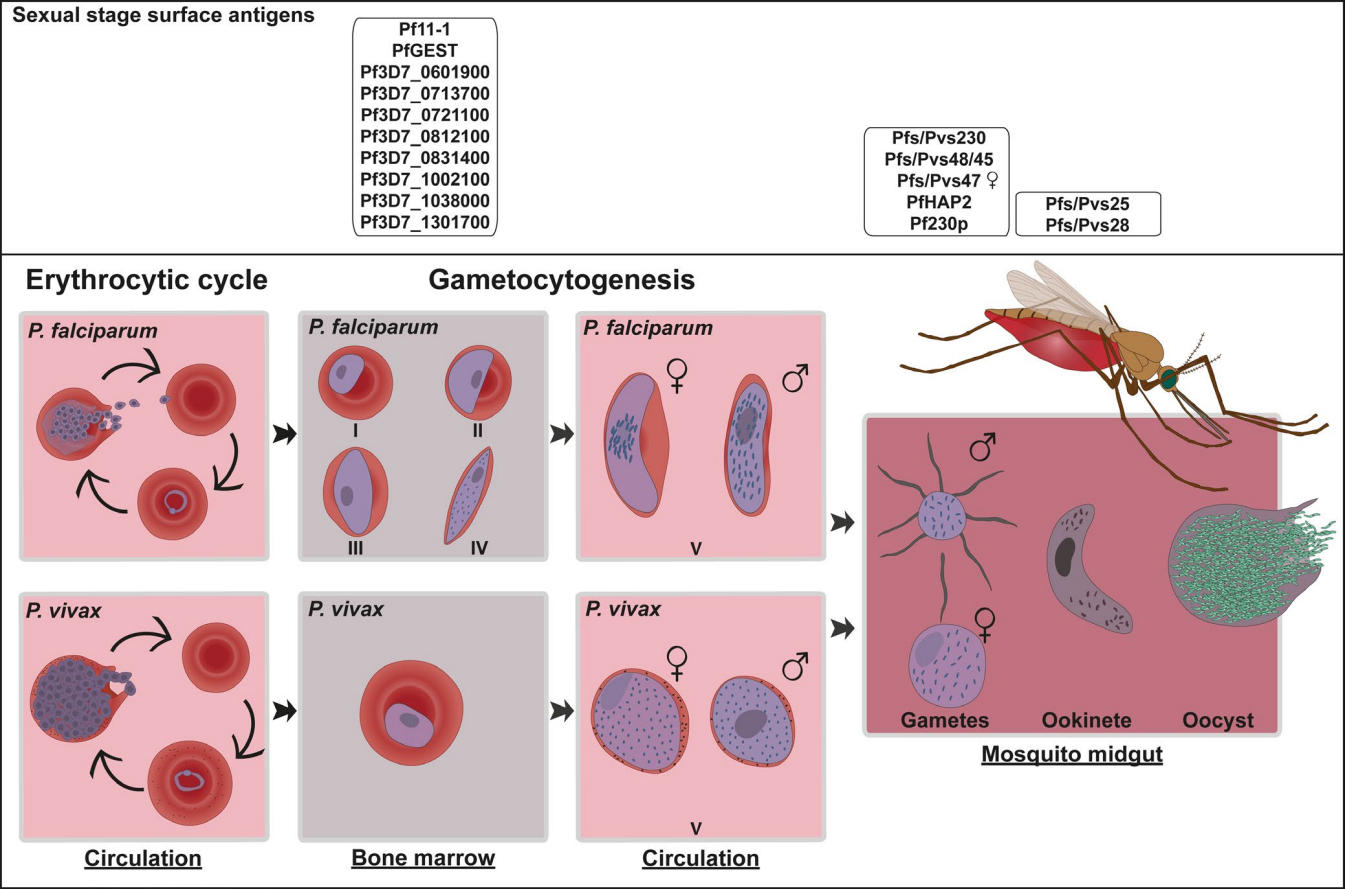
The sexual stage development of *Plasmodium falciparum* and *P. vivax* parasites. Schematic illustration of the development of intra‐erythrocytic gametocytes and post‐transmission development in the mosquito midgut

Gametocytes are the only *Plasmodium* life stages that are infectious to mosquitoes, so the uptake of these specialized forms by blood‐feeding female *Anopheles* mosquitoes is essential for human‐to‐mosquito transmission. *Plasmodium falciparum* gametocytes form when asexual schizonts become committed to produce sexual progeny by the activation and expression of the Apatella2‐g gene (*AP2‐G*).^10^, ^11^ The expression of AP2‐G is under tight epigenetic control by *P. falciparum* heterochromatin protein 1 (PfHP1).^12^ The interplay between histone deacetylases^13^ and gametocyte development 1 (GDV1)^14^ in turn determines the binding or release of PfHP1 and thus the expression of AP2‐G. AP2‐G is a highly conserved member of the apicomplexan AP2 (APiAP2) family of DNA binding proteins whereby its DNA binding domains are highly conserved across all *Plasmodium* species; all *P. falciparum* ApiAP2 proteins have syntenic homologues in *P. vivax* and are expressed at a similar stage of development.^15^ For *P. falciparum*, gametocyte formation is a 10‐12 day process during which the parasite passes through five morphologically distinct forms (stages I‐V) (Figure 1). Immature gametocytes (stages I‐IV) sequester outside the peripheral circulation, primarily in the bone marrow and spleen,^16^ and are released in the circulation to complete their final maturation steps.^17^ The mature stage V gametocytes then become accessible in the peripheral blood for uptake by blood‐feeding mosquitoes.^18^ The development of *P. vivax* gametocytes is markedly faster than *P. falciparum* and only approximately 48 hours are required for maturation^19^ that may also involve a bone marrow phase.^20^ The circulation time of *P. falciparum* and *P. vivax* gametocytes differs significantly. While mature *P. falciparum* gametocytes can be detected for several weeks after clearance of asexual parasites,^21^, ^22^ the half‐life of *P. vivax* gametocyte is very short,^23^ with microscopically detectable gametocytes and gametocyte‐specific mRNA disappearing within days of asexual stage clearance.^23^, ^24^ Stage V *P. falciparum* gametocytes can be morphologically recognized by their characteristic crescent shape, while mature *P. vivax* gametocytes display a round shape and almost fill the entire red blood cell (RBC)^19^ (Figure 1).

In the mosquito midgut, *Plasmodium* gametocytes rapidly egress from the host erythrocyte and develop into gametes. Gametogenesis is induced by a reduction in temperature, increase in pH and exposure to xanthurenic acid.^25^, ^26^ Male gametocytes exflagellate producing up to eight motile microgametes; whereas, female gametocytes “round‐up” to form one immotile macrogamete.^27^, ^28^ Fertilization of a macrogamete by a microgamete results in the formation of a zygote, which then develops into an intermediate “retort” leading to the formation of a mature motile ookinete that traverses the midgut wall and forms an oocyst. Approximately 10‐12 days after blood meal ingestion the rupture of oocysts results in the release of sporozoites, which will invade the mosquito salivary glands completing the mosquito stage of the *Plasmodium* life cycle.^29^

**Figure 2:**
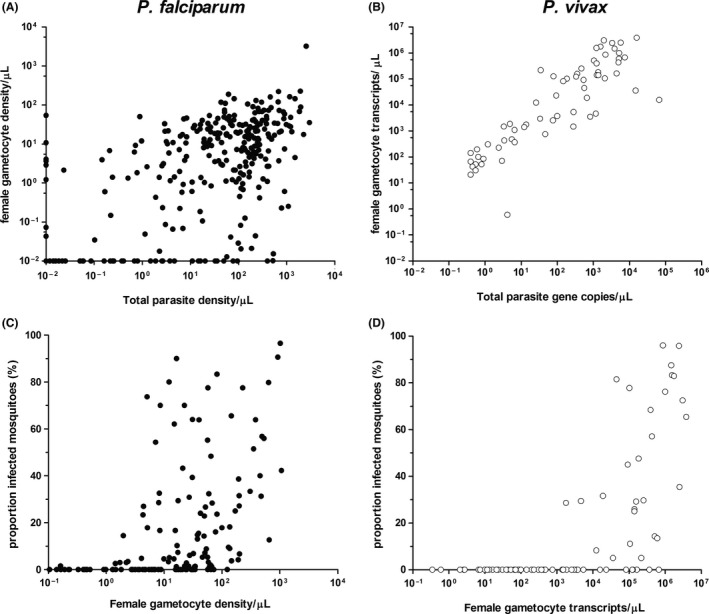
Parasite and gametocyte densities in relation to each other and the proportion of infected mosquitoes. Log_10_ transformed parasite (*X*‐axes) and gametocyte (*Y*‐axes) quantities are indicated for *Plasmodium falciparum* (A) and *P. vivax* (B). Total parasite density is measured using 18S based quantitative polymerase chain reaction (qPCR) and female gametocytes were quantified in reverse transcription‐based qPCR assays that targeted Pfs25 for *P. falciparum* and Pvs25 for *P. vivax*. Indicated are parasite and gametocyte densities/µL for *P. falciparum* and gene copies/µL for *P. vivax*. Parasite and gametocyte culture of NF54 was used for quantification for *P. falciparum*. For *P. vivax* gene copies were quantified from recombinant plasmids containing the respective genes. Log_10_ transformed *P. falciparum* gametocyte density/µL (C) and *P. vivax* transcript copies/µL (D) are indicated in the *X*‐axes with respect to the percent of infected mosquitoes (*Y*‐axes). Data points are indicated in filled circles for *P. falciparum* and unfilled circles for *P. vivax*

Many factors influence the likelihood of gametocytes being transmitted to mosquitoes and establishing a successful mosquito stage infection.^30^ Considerably more work on gametocyte biology and infectivity has been performed for *P. falciparum* than for *P. vivax*, although it is likely that many factors are shared between *Plasmodium* species. General parasite characteristics that have been associated with differences in transmission potential and infectivity include gametocyte density^31^, ^32^, ^33^, ^34^ (Figure 2), concurrent asexual parasite density,^35^, ^36^ ratio of male and female gametocytes,^31^, ^37^ duration of infection,^35^, ^38^ and level of gametocyte maturity.^39^ Host factors such as anemia, age, mosquito factors, and importantly, human immunity are also known to affect gametocyte infectiousness.^40^, ^41^

The first empirical evidence that human immune responses to gametocytes could affect their infectiousness to mosquitoes came from immunization studies in birds,^42^, ^43^, ^44^ following earlier observations that gametocyte infectivity per capita appeared to change the course of an infection.^45^, ^46^ These experiments led to the identification of a small number of proteins expressed by gametocytes, gametes or ookinetes, which for decades have been the focus of gametocyte research and formed the basis of malaria transmission blocking vaccine (TBV) development.^47^, ^48^ Research on gametocyte immunobiology has been outweighed by research on the life stages leading to human infection (the pre‐erythrocytic stages) and clinical disease (the asexual blood stages), but as TBV development has gained pace our understanding of gametocyte biology has improved dramatically. In 2002, the *P. falciparum* genome^49^ and proteome^50^, ^51^ were first published. These and many subsequent investigations have revealed biology that is unique to gametocytes (reviewed by Beri et al^52^), the gametocyte sexes,^53^, ^54^ and different stages of their development.^55^ An integrated approach using proteomic and transcriptomic data from 18 studies predicted 602 proteins to be enriched in *P. falciparum* gametocytes^56^; transcriptome analysis in *P. vivax* revealed that the expression of 1613 genes was correlated with the expression of known gametocyte genes.^57^ Many of the proteins produced specifically by gametocytes remodel the human host cell to support their morphological development,^58^ while others have roles during gametogenesis and fertilization in the mosquito. These proteins represent potential targets of gametocyte‐specific immunity.

This review will discuss the evidence for the existence of naturally acquired human immune responses against the sexual parasite stages of *P. falciparum* and *P. vivax,* discuss the effect of these responses on transmission, and propose strategies for transmission blocking interventions*.* Immature and mature gametocytes have markedly different biology, morphology, and preferential localization in human tissues. Immune responses to early and late gametocytes therefore have the potential to affect transmission differently; early gametocyte immunity could reduce the number of gametocytes achieving maturity in the peripheral blood, while late gametocyte immunity may affect gametocyte number and their likelihood of undergoing successful sporogonic development in the mosquito. While this review will focus on *P. falciparum*, we will also summarize the current state of knowledge for the less‐studied *P. vivax* and indicate major knowledge gaps with regard to anti‐gametocyte immunity, implications for transmission dynamics and potential vaccine strategies.

Merozoites that are released from infected liver cells invade RBCs to initiate the erythrocytic cycle. The sexual development is initiated by a subset of parasites that are committed to produce gametocytes. Immature *P. falciparum* gametocytes sequester outside the peripheral circulation, primarily in the bone marrow and spleen. Their maturation (10‐12 days) involves five distinct developmental stages (I‐V); mature stage V are released in the peripheral circulation. *Plasmodium vivax* gametocytogenesis may also involve a blood marrow phase, but in contrast to *P. falciparum* only takes 48 hours. After ingestion by a blood‐feeding *Anopheles* mosquito, gametocytes rapidly egress from the host RBC and develop into gametes. Male gametocytes exflagellate to form eight microgametes that subsequently fertilize a “round‐up” microgamete to form a zygote leading to the formation of a motile ookinete. The ookinete penetrates the midgut wall and forms an oocyst that produces hundreds to thousands of sporozoites. Upon oocyst rupture, sporozoites are released and migrate to the salivary glands from where they can be transmitted to a new human host. Boxes represent surface antigens that are under consideration for vaccine development.

## 2 IMMUNE RESPONSES AFFECTING GAMETOCYTES IN HUMAN CIRCULATION

### 2.1 Immune responses targeting gametocyte sequestration

Both the asexual and sexual intra‐erythrocytic forms of *P. falciparum* sequester to avoid prolonged circulation in the blood. Asexual parasites sequester in the tissue microvasculature through well‐defined ligand‐receptor interactions; Knob‐associated histidine rich protein (KAHRP) is critical to the formation of “knobs” on the infected erythrocyte surface,^59^ while members of the *P. falciparum* erythrocyte membrane protein 1 (PfEMP1) family accumulate on these knobs and mediate cytoadherence.^60^ The first stages of *P. falciparum* gametocyte development are marked by changes in gene expression. *Plasmodium falciparum* gametocyte exported protein 5 (PfGEXP5) is detectable 14 hours after RBC invasion by a sexually committed merozoite^61^; Pfs16^62^ and Pfg27^63^ are detectable from 24 to 30 hours post invasion. At the same time, proteins associated with asexual stage cytoadherence are down regulated; stage I gametocytes still have a smooth surface without any knobs.^64^ By stage II, gametocytes are morphologically distinguishable from asexual trophozoites, KAHRP protein is undetectable, and PfEMP1 protein is present only at low levels.^64^ Unlike the asexual blood stages, *P. falciparum* gametocytes sequester primarily in the bone marrow and spleen.^16^, ^17^, ^65^, ^66^ The role of PfEMP1 in early gametocyte infected RBC (giRBC) adhesion to the bone marrow vasculature is unclear. Adherence of giRBC to either C32 melanoma cells or human bone marrow endothelial cells has been demonstrated,^67^, ^68^ but a later study did not detect any adherence of gametocyte stages later than I and IIa to C32 cells,^69^ and Silvestrini et al^70^ demonstrated only limited adhesion to a variety of endothelial cells. Recently, adhesion experiments using bone marrow mesenchymal stromal cells demonstrated that immature gametocytes were able to adhere to this cell type via unknown ligands on the giRBC surface.^71^

A study using intravital imaging of *P. berghei* parasites in mice demonstrated direct evidence of homing by merozoites to the extravascular niche of the bone marrow and spleen. Using specific inhibitors, de Niz et al demonstrated that this extravasation (movement from the bone marrow/spleen vasculature to the organ's extravascular space) is mediated via a receptor‐ligand interaction^72^; blocking of P‐selectin alone reduced the accumulation of gametocytes in the bone marrow by 60%. They also provide evidence for invasion of RBC precursor cells in the bone marrow by sexual merozoites. Several theories have been proposed to explain the enrichment of gametocytes in the extravascular space of the bone marrow; eg sexually committed merozoites translocate and gametocytes develop in that space, non‐sexually committed merozoites translocate there and commitment occurs in the extravascular space, or young gametocytes translocate there directly.^73^ The *P. berghei* study supports the hypothesis that a subset of sexual merozoites bearing specific surface ligands home to the bone marrow and spleen, bind the epithelium, move into the extravascular space, and invade erythrocyte precursors (abundant in this niche) to become young gametocytes.^72^ Comparative experiments in the same study using human autopsy material indicate that there is a similar phenomenon possible for *P. falciparum.* These data provide evidence for the presence of surface molecules on sexually committed merozoites (or schizonts containing merozoites) that are involved in cytoadhesion during the process of homing and retention in the bone marrow. During the acute phase of infection, both asexual and sexual infected erythrocytes accumulate in the bone marrow, suggesting that early gametocytes can form in the peripheral blood and may specifically home to the bone marrow/spleen. The targeting of any parasite ligands that mediate gametocyte sequestration by immune factors could possibly inhibit transmission potential.

Numerous studies have shown that immature giRBCs are rigid and that a change in host cell deformability occurs in the transition to maturity.^58^, ^74^ Interestingly, the study by de Niz et al showed that mature *P. berghei* gametocytes pass freely into and out of the vascular spaces of the bone marrow, and that a switch in host cell deformability (here tested by blocking the signal cascade leading to host cell deformability) underlies this freedom of movement.^72^ This supports the hypothesis that gametocyte sequestration is maintained not by receptor‐ligand interactions, but by mechanical retention. Additionally, the flexibility of mature gametocytes allows them to transit the splenic endothelial slits and thereby escape clearance. Members of the STEVOR protein family are associated with the erythrocyte membrane of immature gametocytes. Accompanying the shift in deformability as gametocytes mature STEVOR disappears, indicating a possible role for this protein family in this process.^58^

Naturally induced antibodies may affect gametocyte morphology and fitness. In one study, serum antibodies from Thai malaria patients were incubated with stage I gametocytes, and were observed to reduce their numbers, interfere with maturation, distort their morphology and reduce the number of oocysts developing in subsequent mosquito feeding assays.^75^ Here, the binding of antibodies to the surface of immature forms from stage II onwards was described as a possible mechanism for the observed transmission reduction. Immune responses against antigens on the immature giRBC could affect gametocyte development or circulation time by interfering with sequestration or mediating direct clearance. An epidemiological study performed in Indonesia in the early 1990s compared two groups living in an hyperendemic area; native residents and transmigrants with no (or limited) history of malaria. Lower gametocyte densities in native residents were attributed to specific immune responses,^76^ giving rise to the hypothesis that naturally acquired antibodies against surface antigens on giRBC may directly affect gametocyte densities in circulation independent of a reduction in the asexual parasite biomass.

Several studies have aimed to identify the erythrocyte surface antigens of immature gametocytes that could be involved in sequestration. The first study used a flow cytometry‐based method with purified *P. falciparum* 3D7 gametocytes, and observed reactivity of immune sera from Gambian children with the surface of mature giRBCs, but not with immature stages.^77^ No association was observed in antibody recognition of asexual parasites and mature gametocytes, indicating that a distinct antigen panel is displayed on giRBCs. Follow‐up data on gametocytemia also suggested that antibodies against giRBCs might be able to control gametocyte densities. A more thorough study over a 5‐week period in a Ghanaian cohort also demonstrated the presence of antibodies against mature 3D7 giRBCs using flow cytometry.^78^ These findings were confirmed by repeating the experiments using two clinical Kenyan isolates. In antibody staining experiments analyzed using microscopy, no antibody reactivity was observed against immature giRBCs.

Chan et al quantified antibody reactivity to erythrocytes infected with gametocytes and asexual stages using microscopy in order to better understand the difference in humoral response against these two life stages.^79^ Among two Kenyan cohorts, low antibody reactivity was observed against stage II to V giRBCs from the 3D7 strain. This low reactivity is contrasted with high antibody responses to the surface of trophozoite infected erythrocytes. To confirm their hypothesis that these high responses are the result of reactivity to PfEMP1 the experiment was repeated with two transgenic parasite strains with repressed PfEMP1 expression. In the absence of PfEMP1, surface reactivity of erythrocytes infected with asexual parasites was equal to giRBCs, indicating that PfEMP1 is the major asexual stage erythrocyte surface antigen. The low levels of PfEMP1 expression and the absence of other immunogenic antigens on the surface of giRBCs would explain the observed low antibody reactivity.

Interestingly, a recent study demonstrated that surface recognition by naturally acquired antibodies was only present on erythrocytes infected with immature forms (I to III) of NF54 and a genetic strain with a Pf2004 background. In this study, no measurable reactivity with the surface of giRBC infected with mature stage V gametocytes was observed.^80^ The authors emphasized that their contrasting findings could be due to the more stringent conditions in obtaining the different developmental gametocyte forms, with which RBC integrity and the activation of mature gametocytes into gametes were controlled for by counterstaining with antibodies specific to proteins on the gametocyte (not giRBC) surface. The authors used a transgenic parasite line and flow cytometry to demonstrate reactivity of the Malawian immune sera to antigens on the surface of immature giRBC, that are mostly shared with asexual infected erythrocytes. Subsequently, they used three complementary approaches to identify the antigenic targets. First, they probed giRBC membranes (stage I to III) with Malawian immune plasma to identify differential protein bands between surface‐intact and surface‐depleted samples using mass spectrometry. Additionally, they probed sera of mice immunized with the membranes used for mass spectrometry on a protein microarray consisting of gametocyte proteins.^81^ Lastly, they used the same protein microarray to construct an immune profile for a selection of the plasma samples that showed a range of membrane reactivity as identified using flow cytometry. Combining data of these three approaches and an initial filtering resulted in an overlapping list of 30 proteins of which 26 are predicted to be exported. The vast majority of these hits were shared with asexual life stages. Responses to giRBC were associated with increased phagocytosis of erythrocytes infected with both asexuals and gametocytes. This suggests a possible mechanism of increased clearance of giRBC by antibody‐mediated phagocytosis. This study provides evidence for the presence of antigens on the surface of erythrocytes infected with immature gametocytes that are targeted by functional naturally acquired responses.

The functional phenotype of giRBC immunity may be related to interference with gametocyte sequestration or clearance of developing gametocytes. Based on the *P. berghei* model it seems likely that a subset of merozoites directly translocate into the bone marrow, spleen, and possibly other areas of low vascular flow, but it is also possible that some degree of homing occurs by early gametocytes.^72^ Inside the bone marrow, adhesion of giRBCs to mesenchymal cells is observed,^71^ indicating the presence of early giRBC surface antigens. For inhibition of sequestration, targeting the initial homing of these tissues is a plausible transmission blocking strategy. Alternatively, antibodies may neutralize gametocytes that are developing in the bone marrow. Both interference with sequestration and neutralization of developing gametocytes would result in a reduced release of mature gametocytes into the circulation. As such, giRBC immunity may contribute to the variation that is observed among natural infections in the production of *P. falciparum* mature gametocytes (Figure 2A), although this association remains to be established. Despite remarkable differences in gametocyte development between *P. falciparum* and *P. vivax*, it has been demonstrated that there are similarities in sexual stage gene expression dynamics,^20^ suggesting conservation of pathways involved in sexual development. Erythrocytes infected with *P. vivax* parasites lack knob structures, so it was thought that ligand‐receptor mediated sequestration was not possible for this species. However, it has been shown in several in vitro studies that *P. vivax*‐infected erythrocytes can adhere to a variety of cells including lung and brain endothelial cells.^82^, ^83^ Furthermore, it has been demonstrated in human^84^ and non‐human primate^20^ biopsies that *P. vivax* gametocytes sequester in the parenchyma of the bone marrow. Whether the *P. berghei* model of invasion followed by gametocyte development in the extravascular niche^72^ also applies to *P. vivax* remains to be seen. In contrast to *P. falciparum, P. vivax*‐infected erythrocytes are deformable throughout all stages.^85^ This suggests that homing and retention are not mediated by membrane flexibility, but via ligand‐receptor interactions. Interestingly, *P. vivax* parasites lack a homologue of PfEMP1, although they express a group of variable proteins (VIR)^86^ that have been implicated in tissue adhesion.^82^, ^87^ So far there have been no reports of naturally acquired immune responses against these proteins that potentially inhibit sequestration. The tight association between total parasite density and gametocyte density in *P. vivax* (Figure 2B) may argue against an important role of giRBCs immunity in affecting gametocyte production among naturally infected individuals.

### 2.2 Immune responses influencing gametocyte tropism

Mature gametocytes are ingested during a mosquito blood meal from the sub‐dermal capillaries. Convincing evidence that gametocytes accumulate preferentially in capillary beds is lacking^88^; however, one hypothesis is that mature giRBC surface antigens bind these tissues specifically (“tropins”) or facilitate their release from sequestration or visceral circulation at times when mosquitoes are feeding (“circadins”).^89^

STEVORs, RIFINS, and SURFINs are all hypothetical mature giRBC surface antigens, but as yet none have been shown to mediate gametocyte tropism in the sub‐dermal capillaries.^89^ Though there is limited consensus in prior studies,^66^, ^67^, ^68^, ^69^ recent work indicates that there is a progressive loss of giRBC surface recognition by immune sera during gametocyte maturation.^80^ The lack of antigens on the surface of erythrocytes infected with mature gametocytes is a plausible mechanism of immune evasion, and there is a hypothesis that the specific crescent shape of *P. falciparum* mature gametocytes may be sufficient to result in their disproportionate accumulate in the capillary beds.^90^ Obviously, this cannot be the case for *P. vivax* gametocytes that are transmitted efficiently despite their spherical morphology. At present, there is therefore no evidence for the existence of “tropins” or “circadins” or associated immune responses that could affect gametocyte densities in the skin.

### 2.3 Cellular immunity affecting circulating gametocytes

Although to a limited extent, anti‐malaria cellular and innate immunity might play a role in reducing malaria transmission to mosquitoes. It is well‐established that merozoites and erythrocytes infected with asexual parasites are phagocytosed by monocytes and neutrophils.^91^, ^92^, ^93^, ^94^ The internalization of non‐opsonized infected erythrocytes by monocytes and culture‐derived macrophages is mediated by interactions of CD36 and the parasite ligand PfEMP1.^93^ There is evidence that erythrocytes infected with stage I and stage IIa *P. falciparum* gametocytes are phagocytosed in a similar way.^95^ Phagocytosis of giRBCs later than stage II has not been demonstrated.^95^, ^96^

The role of cellular immune mechanisms in the clearance of circulating gametocytes is contentious. As erythrocytes lack major histocompatibility complex molecules, direct targeting of giRBCs by T lymphocytes is not possible. However, CD4 + T cells clearly respond to gametocyte antigens^97^, ^98^, ^99^ and appear capable of inducing long lasting gametocytocidal immunity in rodent models.^100^ Serum factors from splenectomized macaques (infected with *P. cynomolgi*) taken at the point of infection “crisis” or paroxysm can kill gametocytes, and this appears to be mediated by inflammatory cytokines including tumor necrosis factor‐α (TNF‐α) which stimulates leukocytes to produce toxic nitric oxides.^101^, ^102^ In semi‐immune *P. vivax*‐infected humans, cytokine concentrations were insufficient to induce killing factors during paroxysm,^103^ and it is unclear how this may differ in non‐immune humans. Interestingly, gametocyte killing factors appear non‐specific to species or parasite stage; that is, the supernatant from peripheral blood mononuclear cells stimulated with *P. vivax* schizont extract was able to kill *P. falciparum* gametocytes, and *vice versa*.^102^ T cell responses seem similarly non‐specific to parasite stage.^104^ These data present an exciting avenue for whole parasite vaccine development,^105^ but it remains unclear whether infection crisis in humans leads to meaningful levels of gametocyte death.

## 3 IMMUNE RESPONSES AGAINST INTRA‐ERYTHROCYTIC GAMETOCYTES AND EXOERYTHROCYTIC GAMETES

In contrast to the sparse and partially conflicting evidence for immune responses against intact giRBCs that may reduce circulating gametocyte density, there is a large and cohesive body of data demonstrating that humoral responses to intra‐erythrocytic gametocyte proteins can inhibit parasite development inside mosquitoes.^44^, ^81^, ^106^, ^107^, ^108^, ^109^, ^110^, ^111^, ^112^, ^113^, ^114^, ^115^, ^116^, ^117^, ^118^, ^119^, ^120^, ^121^, ^122^ The antigens responsible are not present on the erythrocyte surface but are expressed on the intra‐erythrocytic gametocyte during their maturation in humans. Key gametocyte surface antigens shared by gametes are involved in processes necessary for colonization of the mosquito midgut: egress from the RBC; male gamete exflagellation and exflagellation center formation; fertilization and ookinete invasion of the gut epithelium. As gametocytes die in the human host they are cleared by the spleen and the immune system is exposed to these antigens that are shared by the human and mosquito parasite stages. The resulting antibodies circulate in humans, but their functional consequence only becomes apparent in the mosquito, where they can interfere with parasites in the mosquito midgut and cause inhibition or total arrest of the mosquito infection. Though the effect is likely to be transitory and quantitatively less profound compared to transmission inhibition, there is also some evidence that immune factors may lead to enhancement of gamete infectivity in certain conditions.^123^

The proof of principle for the existence of transmission reducing (TR) antibody responses comes from experiments in which birds were immunized with whole inactivated gametocytes or gametes in the 1950’s and 1970’s.^42^, ^43^, ^44^, ^106^, ^124^ Evidence for naturally acquired TR immunity acting to prevent mosquito stage parasite development comes from cross‐sectional studies using mosquito feeding assays. Mendis et al^109^ showed that Sri Lankan individuals with acute *P. vivax* infections produced gamete specific antibodies, and that antibodies from these patients inhibited transmission in direct membrane feeding assays (DMFA). Shortly after, Graves et al showed that similar mechanisms prevented *P. falciparum* gamete viability using the standard membrane feeding assay (SMFA), in which the effect of sera from patients in Papua New Guinea was tested on cultured gametocytes.^108^ These experiments demonstrated that the dominant immune mediator of gametocyte infectivity appears to be the humoral immune response. Antibodies directed to surface antigens on the gamete surface may prevent fertilization via direct lysis of gametes by activation of the complement system,^125^, ^126^ opsonization resulting in immune cell‐mediated lysis^127^ or agglutination of gametes.^128^, ^129^

Parasite antigens that can be targeted by antibodies to inhibit transmission can be divided into two broad classes; prefertilization and the postfertilization antigens. Prefertilization antigens are expressed during gametocyte development and contribute to the viability of mosquito stage gametes, zygotes and/or ookinetes. Antibodies to prefertilization antigens are naturally acquired in the human host only because the majority of gametocytes die in circulation, releasing their intra‐erythrocytic proteins. Postfertilization antigens are expressed solely in the mosquito vector; though transcription may occur in circulating gametocytes, the resulting mRNA is held in translational repression until the gametocytes activate in the mosquito midgut.^130^ Consequently, humans do not acquire humoral responses to these antigens. This second group of antigens is beyond the scope of this review, since no naturally acquired humoral responses are observed to these antigens.

The most studied prefertilization antigens are P48/45 and P230, which belong to the 6‐cysteine protein family^131^ and play crucial roles in fertilization.^132^, ^133^ These proteins were first identified as targets of transmission blocking monoclonal antibodies (mAbs) isolated from mice immunized with gametocyte/gamete preparations.^48^, ^107^ The first evidence of naturally acquired antibody responses to these proteins was shown in sera from individuals from Papua New Guinea.^108^ P48/45 is attached to the surface of both female and male gametes via a glycophosphatidylinositol‐anchor and forms a stable complex with P230.^134^, ^135^, ^136^ Male gametes of Pfs48/45 and Pbs48/45 knock‐out lines are unable to adhere and penetrate female gametes, which results in a dramatic reduction of oocyst number.^132^ Although P48/45 is expressed on the surface of macrogametes, its disruption in female gametes does not seem to affect their fertility. Replacing the endogenous *Pb48/45* by its vivax orthologue seemed to reduce oocyst development, but did not abolish it, indicating that there is probably functional conservation of P48/45 in these two parasite strains since fertilization was not affected.^137^ In *P. falciparum* gametocytes, Pfs230 is present on the gametocyte surface, and a 50 kDa fragment is proteolytically cleaved from the surface‐bound protein after the parasite’s emergence from the RBC in the mosquito gut.^138^, ^139^ Transgenic *P. falciparum* Pfs48/45 knock‐outs (KO) produce Pfs230, but it is not retained on the gametocyte surface, indicating that Pfs48/45 mediates Pfs230 retention.^133^ Male gametes lacking Pfs230 are still able to undergo host cell egress and exflagellate but are unable to bind uninfected RBCs to form exflagellation centers. The importance of this protein in fertilization is reflected by the significantly reduced oocyst numbers of the Pfs230 KO parasites.^133^

In alignment with their exposure on the surface of gametes and importance in gamete fertility, studies have shown that the presence and titer of naturally acquired antibody responses to Pfs48/45 and Pfs230 are statistically associated with serum transmission reducing activity (TRA).^108^, ^112^, ^113^, ^118^, ^120^, ^140^ Though the TRA of mAbs against Pfs48/45 and Pfs230 was demonstrated decades ago,^47^, ^48^, ^107^ it was only recently shown that naturally acquired antibodies against Pfs48/45 and Pfs230 are functionally involved in natural TRA.^81^ Antibodies against Pfs48/45 and Pfs230 were separately purified from six individuals whose antibodies (total IgG) showed high TRA in the SMFA. The purified antigen‐specific IgGs were reconstituted to the original serum volume and tested in the SMFA. The α‐Pfs48/45 antibodies of one individual blocked transmission independently, whereas α‐Pfs230 antibodies from another donor reduced transmission significantly. Concentration of antibodies resulted in higher TRA for several donors. This was the first direct evidence of the reducing potential of naturally acquired antibodies against Pfs48/45 and Pfs230.

Besides P230, rodent and human parasites also encode a paralog P230p. This protein is only expressed by mature male gametocytes.^141^ Disruption of the gene in *P. berghei* did not result in any defects throughout the life cycle, indicating that the protein is dispensable.^142^ The *p230p* locus in *P. berghei* 143, 144, 145 and *P. knowlesi* 146 parasites has been commonly used as a neutral insertion cassette to generate transgenic parasites. On the other hand, *P. falciparum* mutants lacking Pf230p have a strongly reduced ability to bind erythrocytes to form exflagellation centers, similar to the observed phenotype in Pfs230 KO parasites.^147^ Pf230p KO resulted in a dramatic reduction in oocyst density in mosquitoes, indicating an important role in fertilization. The misconception that Pfs230p is dispensable has resulted in the disregard of this protein as a target of TR immunity. To our knowledge mAbs targeting P230p in *P. falciparum* or *P. vivax* have neither been tested, nor have proteins been produced for immunization studies with a view to TBV testing. Naturally acquired Pfs230p antibody responses have been assessed in one study, which linked the TRA of serum antibodies from individuals living in malaria endemic areas with the same individual's antibody responses to 315 gametocyte enriched proteins.^81^ Pfs230p was not the focus of this study, but the microarray data generated are publicly accessible (https://doi.org/10.5061/dryad.8bp05). The magnitude of α‐Pfs230p responses was not significantly different between individuals with evidence of blocking transmission in the SMFA and individuals whose antibodies had no notable transmission reduction activity. However, the proportion of individuals deemed “sero‐reactive” in a mixture model was borderline significantly higher in blockers (13.6%) than in non‐blockers (3.9%) for one of the two Pfs230p peptides tested (PF3D7_0208900.e1s2, *P* = .054). It should be noted that this analysis was not adjusted for false discovery from multiple comparisons, and the overall prevalence of responses was low (13/276). Further serological studies with Pfs230p in its native conformation will be valuable.

Pfs47 is another member of the 6‐cysteine protein family^131^ and is a paralog of Pfs48/45. It is specifically expressed in female gametocytes and present on the surface of female gametes, zygotes and ookinetes.^148^, ^149^ The protein is known to protect the ookinete from the mosquito complement system by disrupting the c‐Jun N‐terminal kinase pathway in *A. gambiae*.^150^ However, Pfs47’s role appears unessential as disruption of the gene does not result in a reduction in oocyst numbers in *A. stephensi*.^148^, ^151^ Interestingly, in *P. berghei* the opposite has been demonstrated, with gene disruption resulting in a significant reduction of oocyst numbers.^132^, ^152^ Although Pb47 and Pfs47 are clear paralogs, there is only limited sequence conservation,^153^ which could explain the observed differences in function. There has been contrasting evidence for the effect of mAbs against Pfs47. A study by van Schaijk et al^148^ showed that transmission was not affected by any of the three different Pfs47 specific mAbs in the SMFA. However, recent data suggest that antibodies specific to the central region have the ability to reduce the number of ookinetes in the mosquito midgut and thereby reduce transmission to both *A. gambiae and A. stephensi*.^154^ It remains unresolved whether Pfs47 has an essential role in either fertilization or ookinete protection. The magnitude and prevalence of naturally acquired antibodies to Pfs47 appear nearly identical in individuals with antibodies that block transmission in the SMFA and those without such antibodies.^81^

The observed differences in phenotype between *P. berghei* and *P. falciparum* after disruption of *P230p* and *P47* suggest that there are functional differences in these proteins between these two *Plasmodium* spp. There are no data available with respect to the function of these proteins in *P. vivax,* and it remains unknown whether they have an essential role in mosquito stage development.

The male‐specific sterility gene (*HAP2*) was first identified in *Arabidopsis thaliana*,^155^ and HAP2 homologues were later identified in higher plants and protists, including *P. berghei*.^156^ In *P. berghei* HAP2 is expressed in gametocytes and is present on the surface of intra‐erythrocytic gametocytes and microgametes. Disruption of the gene results in reduced transmission by blocking gamete fertilization.^157^ Using membrane dyes this report demonstrated that HAP2 is not involved in adhesion of gametes but plays a role in membrane fusion during zygote formation. Serum from rodents immunized with recombinant PbHAP2^158^ or PfHAP2^159^ inhibited oocyst development in the SMFA. Naturally acquired antibodies against the recombinant PfHAP2 were identified in sera from Malian adults^159^; however, it remains unclear if these functionally contribute to TRA in the field. No significant differences in PfHAP2‐specific antibody magnitude or prevalence were observed between SMFA blockers and non‐blockers in microarray analyses.^81^ However, it has been recently demonstrated that sera from mice immunized with peptides targeting the fusion loop of PfHAP2 inhibit the transmission of *P. falciparum* gametocytes sourced from naturally infected donors.^160^ These data make HAP2 an interesting antigen for more extensive study. To our knowledge there are currently no data testing HAP2 function in *P. vivax,* though it seems likely that the function is conserved in *P. vivax* based on the observed functional conservation of HAP2 throughout species.^156^

Antibody‐mediated transmission reduction has been observed in the absence of Pfs48/45 and Pfs230 antibodies in serum,^41^, ^108^, ^110^, ^114^ and after active depletion of these antibodies from purified total IgG fractions.^81^ These observations have led to the hypothesis that antibody responses to other sexual stage pre‐fertilisation surface‐associated proteins may contribute to naturally acquired TRA. In a recent microarray analysis of antibody profiles against gametocytes and TRA, antibody responses to 13 novel proteins were associated with TRA and displayed features that suggest surface expression. This includes Pf11‐1 and PfGEST, which have both been implicated in the process of gamete egress from the erythrocyte.^161^, ^162^, ^163^ Monoclonal antibodies against Pf11‐1 can reduce transmission possibly by interfering with egress.^162^ PbGEST KO gametes show a clear defect in host cell egress^163^; however, there are no data on the *P. falciparum* orthologue. Future work should include the evaluation of these antigens in rodent immunization studies to determine their potential to induce functional antibody responses. The remainder of the TRA associated antibody specificities in this analysis included largely conserved proteins with an unknown function, so it remains to be seen if these have the potential to induce functional transmission blocking immunity.

Though antibodies targeting gametocytes has been the focus of epidemiological and vaccine focused research, other human immune factors are ingested by mosquitoes when they feed and co‐circulate with parasites as they activate and develop in the mosquito gut. Human phagocytes are present in the predigested blood meal and gametes (after RBC egress) are potentially vulnerable to direct phagocytosis. It has been shown in vitro that extracellular gametes can be phagocytosed and that the addition of immune serum leads to an increase in phagocytosis.^94^ However, these processes were inefficient in the environment of the mosquito midgut, probably due to the reduced temperature (26°C rather than 37°C). The role of human cellular immunity on mosquito stage parasites is likely to be limited.

## 4 MODULATION OF TRANSMISSION BY ANTI‐GAMETOCYTE IMMUNE RESPONSES

### 4.1 Read‐outs and methods of assessment

Immune modulation of malaria transmission can be assessed directly using mosquito feeding assays,^108^, ^123^ which can determine both the direct transmission potential of naturally infected hosts and the effect of host immune factors. The SMFA is the most controlled of these assays, in which plasma, serum or their purified components are added to a blood source containing cultured gametocytes.^164^ Multiple SMFA feeds can be performed in parallel with the same infective material, and all experiments are performed with a relevant control (eg the same infective material with plasma from non‐malaria endemic areas) allowing results to be combined and compared reliably between experiments. Transgenic parasites can be used to increase the scalability of the SMFA for population cross sectional screening, using luminescence as the assay read‐out rather than dissection and oocyst counting.^165^ Alternatively, the DMFA allows the impact of immune factors to be assessed on locally circulating parasite strains with more natural gametocyte characteristics (ie density, sex ratio, maturity). In its most basic form, the DMFA involves feeding colony adapted mosquitoes with blood collected from naturally infected individuals to determine their transmission potential; as in the SMFA, the traditional read‐out is the number of midgut oocysts or salivary gland sporozoites. To assess immune modulation, the plasma component of the blood sample (ie its autologous plasma) can be replaced with naive plasma, revealing the transmission potential of gametocytes in the absence of immune factors from the host. In this “serum‐replacement” version of the assay (so called because anticoagulants were not used when the assay was developed^109^) transmission modulation due to the autologous plasma is determined by comparison to a feed where autologous plasma is removed and then replaced as a control for the methodological disturbance.^166^

The read‐out of the SMFA and serum‐replacement DMFA is generally reported as percent TRA. This is the percent inhibition of oocyst density (or sometimes prevalence) in test mosquitoes relative to the experimental control. For example, if mosquitoes that have fed on a blood meal containing test plasma results in a mean oocyst load of ten, and control mosquitoes have a oocyst load of 100, the percentage TRA would 90%. Transmission inhibition by immune factors is referred to as either transmission reducing (TR) immunity or transmission blocking (TB) immunity. Specifically, “blocking” should refer only to the total annulment of mosquito infection. Transmission enhancing (TE) immunity is less commonly reported than TR immunity, and though random variation of mosquito infection rate around the baseline (control) is likely to be the cause of many observations of low level TE, there is substantial evidence for immune mediated enhancement of immunity.^167^ TE would be reported as a negative TRA, or a relative infectivity >100%. As with all biological systems, the results of feeding assays require confirmation; replicable TRA of ≥90% in the SMFA is viewed as high level reduction (equivalent to blocking in natural infections).^168^ The aim of TBV development is to induce TB immunity, which will reduce the number of infected mosquitoes feeding on a vaccinee. TR and TE immunity are a continuum (thus transmission modulation) while TB immunity is likely to be uncommon in nature, so we use the former terms.

### 4.2 *Plasmodium falciparum* transmission modulating immunity

In experiments with sera from Papua New Guineans performed by Graves et al, serum TRA in SMFA experiments varied from −124% TRA (TE immunity) to 99.4% (TR immunity). TE immunity was apparent in 10/41 sera (5/33 tested in duplicate).^108^ Subsequent studies have shown that TR immunity against *P. falciparum* develops rapidly after malaria exposure^119^ and is short lived.^120^, ^122^ Cross sectional analyses show wide variations in the frequency and intensity of *P. falciparum* TR immunity,^110^, ^114^, ^121^ which is likely related to differences in sampling strategy and transmission intensity. Van der Kolk et al^121^ performed a rigorous assessment of transmission modulation in 642 sera from Cameroonian, Indonesian, and Tanzanian *P. falciparum* gametocyte carriers. SMFA showed that TR immunity was present in 48% of sera, while TE immunity was present in 7%. The reproducibility of these results was variable, but significant numbers retained their TE and TR activity in repeat feeds. For *P. falciparum*, studies with serum‐replacement DMFA generally show increases in mosquito infection rates between 14% and 66% in the absence of host immune factors,^114^, ^116^, ^169^, ^170^ corroborating the results of individual studies that TR immunity is more common and has a greater effect size than TE immunity.

There is evidence that TR immunity is associated with the presence or titer of antibodies against mature *P. falciparum* gametocyte antigens.^81^, ^108^, ^110^, ^111^, ^112^, ^113^, ^114^, ^115^, ^116^, ^117^, ^118^, ^119^, ^120^, ^121^, ^122^, ^171^, ^172^, ^173^ A synthesis of data from six studies which measured antibody‐mediated TRA in the SMFA and measured α‐Pfs48/45 and α‐Pfs230 responses by enzyme‐linked immunosorbent assay (ELISA)^115^, ^116^, ^118^, ^119^, ^120^, ^121^ showed that there was a significantly increased likelihood of strong TRA (≥90%) for individuals seropositive for either antigen (combined odds ratio [OR] = 3.72 [1.96‐7.15, *P* < .0001]).^41^ Despite the general consensus, several individual studies showed no association between α‐Pfs230 and/or α‐Pfs48/45 and TR immunity.^108^, ^110^, ^114^, ^121^ TR immunity has been observed in the absence of Pfs48/45 or Pfs230 antibodies, and vice versa.^108^, ^113^, ^114^, ^115^, ^120^, ^121^ These somewhat inconsistent findings suggest that TRA due to α‐Pfs48/45 and α‐Pfs230 antibodies is incomplete in most individuals, may be synergistic, and that responses to unknown gametocyte surface proteins may also affect transmission. With an agent based model combining DMFA, antibody, and parasite density data, Ouédraogo et al recently demonstrated that antibodies against Pfs48/45 and Pfs230 were associated with up to 44% reduction in the proportion of infected mosquitoes and up to 70% reduction in oocyst density, among individuals with and without observable gametocytemia in Burkina Faso.^171^ Stone et al^81^ used plasma from numerous cross‐sectional studies^32^, ^35^, ^116^, ^174^, ^175^, ^176^, ^177^, ^178^, ^179^ to assess antibody‐mediated transmission modulation. As in previous studies, antibody‐mediated TRA (quantified in SMFA) was statistically associated with the presence of Pfs48/45 and Pfs230 antibodies (combined OR 5.90 [95% CI: 2.1‐16.7], *P* = .001). TR immunity correlated with antibodies against these en others antigens (including α‐Pf11‐1 and α‐PfGEST) were also associated with reduced infectivity in field based DMFA. *P. falciparum* gametocyte immune responses may therefore contribute to the substantial variation in mosquito infectivity from natural gametocyte donors (Figure 2C,D).

### 4.3 *Plasmodium vivax* transmission modulating immunity

In 1987, Mendis et al showed that naturally acquired antibody‐mediated immune responses to the sexual stages of *P. vivax* (confirmed using immunofluorescence) were able to block *P. vivax* gametocyte transmission in the DMFA.^109^ In this study, two‐thirds of serum samples from acutely infected individuals in Sri Lanka (n = 40) mediated mid to high level reduction of autologous parasite transmission, while three samples showed oocyst intensities in the presence of the test sera “considerably greater than in controls”. In a subsequent study, the authors showed that the effects of this immunity were short‐lived; reductions were associated with an interval of <4 months between a first and second infection.^180^ Also, testing individuals with acute *P. vivax* infection, Carter and Mendis reported complete suppression of mosquito infection by 22% of sera from Sri Lankans (n = 196), and enhancement of infectivity in 12% of sera.^181^ A technically similar study by Ramsey et al showed that Mexican individuals with secondary *P. vivax* infections either completely blocked transmission or showed varying levels of reduction (n = 41). Individuals with primary infections produced a similar proportion of enhancing and reducing effects in the serum‐replacement DMFA (n = 63)*.* 182 TR immunity has been substantiated by numerous other studies^183^, ^184^ and is often accompanied by observations of enhancement.

Few studies assessed the presence of antigen‐specific *P. vivax* sexual stage antibodies,^185^, ^186^ and to our knowledge, none have associated specific antibody responses with TR immunity. This is likely due to difficulties expressing the proteins in significant quantities in correct conformation, and because the focus of sexual stage protein production efforts has been on *P. falciparum*. Various studies however have assessed *P. vivax* gamete recognition using immunofluorescence assays; Mendis^109^ showed that there is a negative correlation between TRA and anti‐gamete antibody titer, while Ranakawa^180^ showed this correlation only when mosquitoes were fed directly on patient blood, but not on the same blood source through a membrane feeder. A comparative study of *P. vivax*‐infected (n = 105) and uninfected individuals (n = 44) from Colombia also examined antibody titer and TRA using serum‐replacement DMFAs. Among infected individuals, 44.6% had 50%‐89% TRA and 35.2% had ≥90% TRA. The correlation between anti‐gamete antibody titre and TRA was clear in exposed, currently uninfected, individuals; individuals with low titres tended to enhance transmission, and individuals with higher titres either had no effect or blocked transmission.^184^

### 4.4 Evidence for transmission enhancing immunity

We recently reviewed the evidence for immune enhancement of *Plasmodium* transmission.^167^ In longitudinal assessments of *P. cynomolgi* infection in macaques, anti‐gamete antibody titer was shown to increase steadily from baseline, the peak level coinciding with a period of serum TR immunity.^187^, ^188^ When the antibody concentration was lower (in the early phase of the infection, and after the peak during convalescence) TE immunity was observed, before eventually both antibody titer and relative infectivity returned to baseline. These findings mirrored observations that gametocytes appeared most infectious to mosquitoes at the very start of a blood stage infection.^189^, ^190^ A key study by Peiris et al showed that dilution of *P. vivax* anti‐gamete antibodies in *P. vivax*‐infected human blood led to enhanced oocyst infection in mosquitoes compared to controls; highly dilute immune serum and mAbs (which caused significant TRA at higher concentrations) promoted infection in experiments where gametocyte density was insufficient to cause infection without additional factors.^123^ In the aforementioned study of *P. vivax* exposed individuals from Colombia, sera with varying levels of TRA were titrated to study the effect of dilution on transmission modulation.^184^ Dilution of sera with low TRA showed enhancement (−200%) when diluted, while dilution of sera with no TRA did not have the same effect, indicating a possible role for low levels of blocking antibodies in TE.

These studies collectively promote the hypothesis that ingestion of low sexual stage antibody titers may lead to enhanced mosquito stage infection. The work of Peiris et al^123^ shows that for *P. vivax*, enhancing antibodies have the same specificity as reducing antibodies. Carter and Mendis suggested that antibody‐mediated TE is the result of both low antibody titers and low inherent gametocyte infectivity (ie low gametocyte densities).^181^ This was apparent in the time‐course studies of *P. cynomolgi*,^187^ and stands in contrast to TR immunity that appears to follow intensive, recent gametocyte exposure. The exact mechanisms of enhancement are unknown, but it may be that at low concentration, transmission reducing antibodies targeting shared surface antigens of both male and female gametes, though unable to neutralize the gametes or promote opsonization, may promote fertilization.^123^ In *P. falciparum*, the evidence for enhancement is less clear. Although multiple studies show low level immune‐mediated enhancement for *P. falciparum*,^108^, ^113^, ^121^, ^164^, ^172^ Ponnudurai et al^164^ reported minimal enhancement due to Pfs48/45 and Pfs25 mAbs at low titer, and concluded that the phenomenon was due to experimental variation. Recent studies add weight to the enhancement hypothesis for *P. falciparum*; mAbs against the central peptides of the D2 region of Pfs47 (present on the gametocyte, gamete, zygote and ookinete surface) blocked transmission to mosquitoes, while mAbs against proteins at the regions N‐terminus gave rise to twice the number of midgut oocysts.^154^ The existence and relevance of TE presents an important knowledge gap in our understanding of how anti‐gametocyte immunity influences transmission dynamics.

## 5 MALARIA TRANSMISSION BLOCKING VACCINE DEVELOPMENT

### 5.1 The conserved nature of gametocyte antigens

The conservation of a protein across parasite isolates and species is an indicator for its specialization and functional preservation. Antigenic variation of *P. falciparum* RBC surface antigens results from the constant re‐organization and variable expression of *var* genes.^191^ This gene family encodes the PfEMP1 surface protein that mediates immune evasion and sequestration. Although the *var* genes family is the most intensively studied, the genetic variation in non‐*var* genes is increasingly appreciated. This is largely due to recent sequencing efforts and the availability of sequence data in open access databases (PlasmoDB,^192^ PlasmoView^193^ and Pf3k^194^). A common observation is that gametocyte‐specific genes show higher sequence conservation than asexual blood stage‐expressed genes.

**Figure 3:**
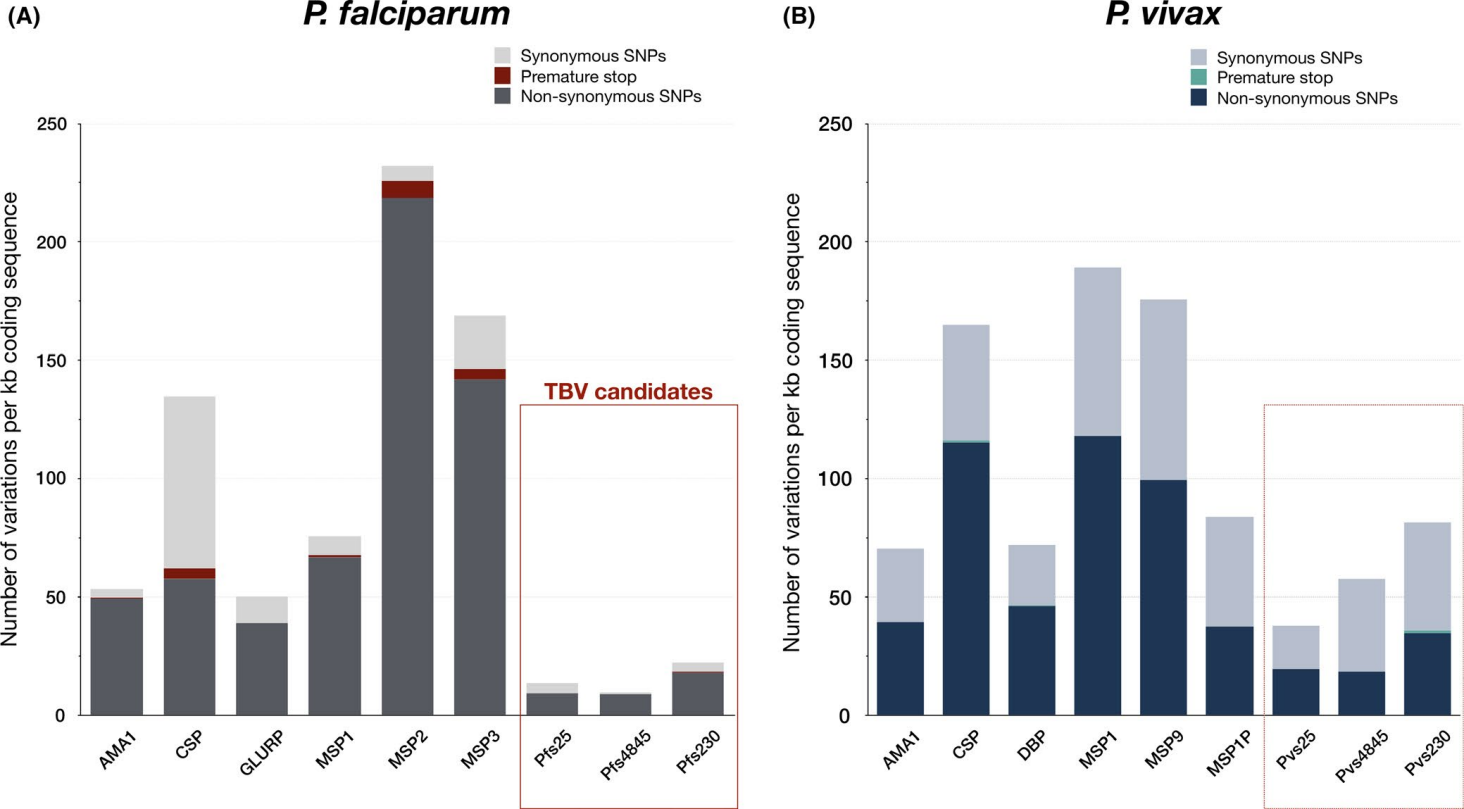
Reported variations in *Plasmodium falciparum* and *P. vivax* vaccine candidates. All plasmoDB (v44) listed variations in *P. falciparum* (A) and *P. vivax* (B) vaccine candidates normalized for gene length. Transmission blocking vaccine (TBV) candidates in *P. falciparum* and their orthologues in *P. vivax* are indicated with a red box and dotted box, respectively. The displayed TBV candidates for *P. falciparum* harbor an average non‐synonymous SNP density of 11.98 SNPs per kb coding sequence, while the pre‐erythrocytic and blood stage candidates have significantly more non‐synonymous SNPs (95.54, *P* = .017, Welch's t test). For *P. vivax*, the orthologues of *P. falciparum* TBV candidates have an average of 24.34 non‐synonymous SNPs/kb, significantly less than the targets of asexual stage vaccine candidates (76.01 non‐synonymous SNPs/kb, *P* = .011, Welch's t test). A premature stop codon is introduced by a non‐synonymous SNP and as a result the stability and function of transcripts and proteins can be altered

Assuming positive selection for variant genes is part of an “arms race” between the human host and the parasite, there are several reasons why gametocyte proteins might be more conserved than blood stage proteins. The human host immune response will be targeted more towards the asexual blood stages as they vastly outnumber gametocytes during acute infection; <5% of microscopically detectable parasites are gametocytes in most endemic settings.^195^ However, this proportion appears to change with host age and immunity, transmission intensity, and duration of infection.^30^ Furthermore, asexual parasites actively remodel the RBC surface protein structure, while mature gametocytes do so to a lesser extent. This difference has implications for the antigenic targets of immunity because cytoplasmic, mitochondrial and nuclear proteins are more conserved than exported or apicoplast‐ and membrane‐targeted proteins.^196^ Recently, the duality between internal and, potentially, secreted proteins was confirmed for immature gametocytes. It was shown that even a subset of surface‐associated early gametocyte proteins had very little sequence variation indicating limited selection by host immunity.^80^ The limited genetic variation in gametocyte‐specific genes is illustrated by challenges to discriminate the gametocytes from multiple clones. The sequences of *Pfs48/45* and *Pfs16* in lab‐adapted strains and isolates from Papua New Guinea showed few polymorphisms, while diversity in *Pfs230* fragments allowed for some discrimination between samples.^197^ Genetic variation among *P. falciparum* TBV candidates and their orthologues in *P. vivax* is lower compared to key vaccine targets for the pre‐erythrocytic or asexual blood stage (*P. falciparum* reviewed in,^198^ *P. vivax* reviewed in^199^) (Figure 3). The high sequence conservation of gametocyte‐specific genes make them attractive targets for vaccination, since this increases the chance of being effective in a strain‐transcendent manner.

### 5.2 Leading transmission blocking vaccine candidates

It is conceptually attractive to develop a vaccine that affects transmission to mosquitoes by inducing immune responses that prevent sequestration, interfere with gametocyte maturation or target mature circulating gametocytes in the human host. Recent findings that antibodies against antigenic targets expressed on the giRBC surface are negatively associated with both asexual and gametocyte load in Malawians^80^ provide a lead for further investigation that should demonstrate whether giRBCs are a viable target for immunization.

By comparison, there is a much longer history of TBV development targeting antigens present on the surface of intra‐erythrocytic gametocytes and exoerythrocytic gametes. The rationale for the development of these TBVs comes from above‐mentioned animal studies where transmission reducing immunity was induced by vaccination^44^, ^106^ and paved the way for the identification of P48/45, P230 and P25 as important TBV candidates.^48^, ^107^

**Figure 4:**
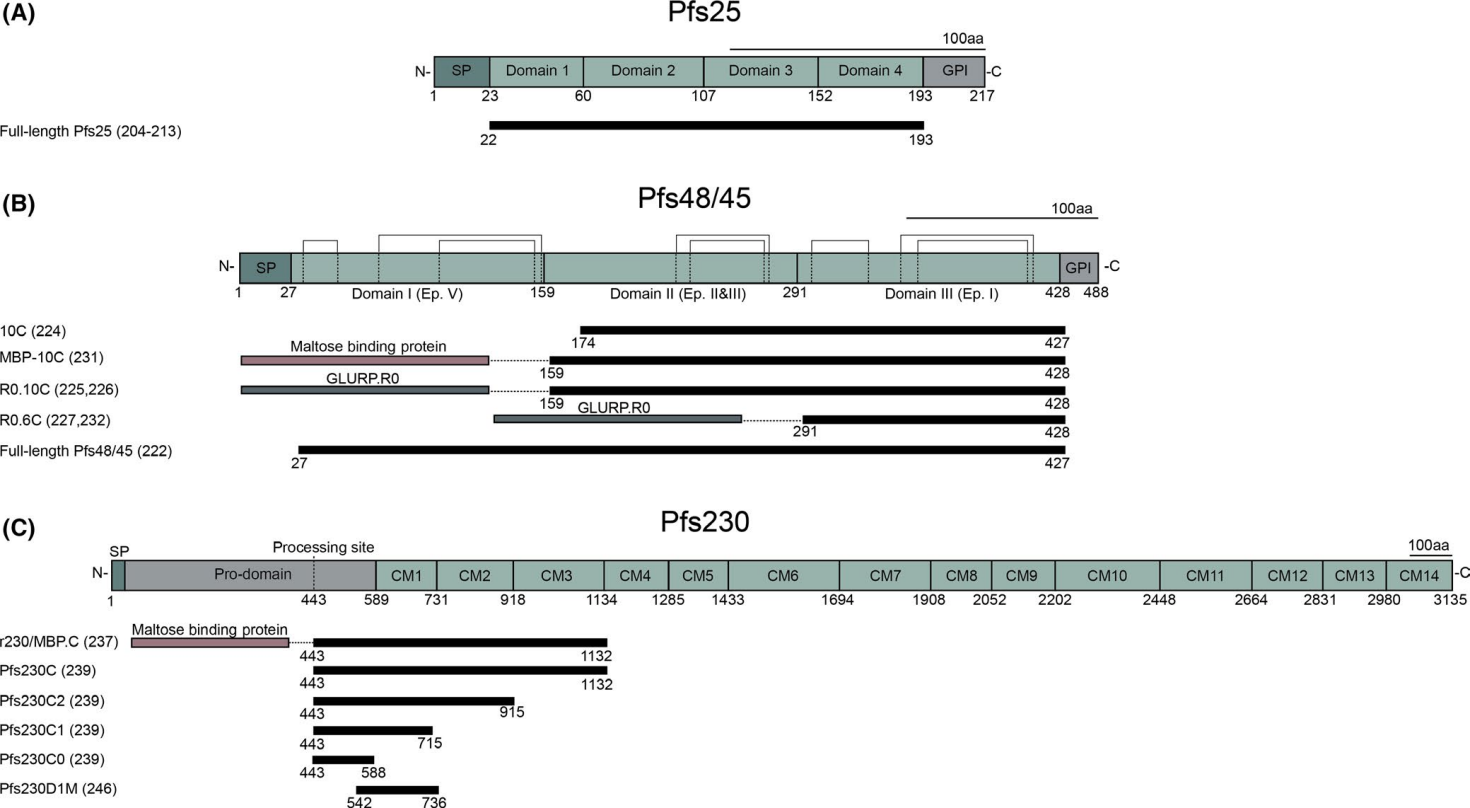
Native protein structure of Pfs25 (A), Pfs48/45 (B) and Pfs230 (C). (A) Schematic representation of the four EGF‐like domains of Pfs25 with 22 cysteines with, underneath, the full‐length vaccine construct used in preclinical and clinical studies. (B) Domain structure of Pfs48/45 with cysteines forming disulphide bridges (dotted lines) based on homology to other 6‐cys domain proteins. Underneath, several vaccine constructs are presented that have been tested in preclinical studies. (C) Schematic of Pfs230 with 14 cysteine motifs (CM). The processing site is the location where the protein is cleaved after gamete emergence from the red blood cell. Underneath, vaccine constructs that have been tested in preclinical studies; Pfs230D1M has been tested in clinical studies (ClinicalTrial.gov NCT02334462 and ClinicalTrial.gov NCT02942277). SP: Signal peptide; GPI: Glycosylphosphatidylinositol anchor

The post‐fertilisation antigen P25 is the most extensively studied TBV candidate to date with a variety of vaccine constructs evaluated in both pre‐clinical studies and clinical trials (reviewed by Chaturvedi et al^200^ and Mueller et al^201^) (Figure 4A). P25 contains four EGF‐like domains with 22 cysteines^202^, ^203^ and is anchored on the surface of zygotes and ookinetes.^48^ The first in‐human trials with full‐length Pfs25 showed modest immunogenicity,^204^ stimulating efforts to develop more potent vaccine products, such as fusion with and coupling to carrier proteins (Pfs25‐EPA,^205^ Pfs25‐IMX313,^206^ Pfs25‐GPI^207^), expression on virus‐like particles^208^ or combining Pfs25 with other adjuvants.^209^, ^210^ Administration of virus‐like particles comprising Pfs25 in healthy US adults was safe but TRA of the induced antibodies was weak.^211^ Pfs25 conjugated to *Pseudomonas aeruginosa* ExoProtein A (EPA) has been tested in malaria naive US adults, and is the only Pfs25 construct tested in malaria‐exposed individuals. Four doses were required to induce higher functional transmission reducing antibody responses in a subset of vaccinated Malian adults compared to the control group and antibody levels waned rapidly.^212^, ^213^ The future of Pfs25 as a TB vaccine is uncertain given the limited efficacy and the availability of other candidates that may hold more promise, including Pfs48/45 and Pfs230.

The development of vaccines against P48/45 and P230 has been challenging due to their complex native conformation. Pfs48/45 is a 51.6 kDa protein and contains three domains with up to six conserved cysteines in each domain^131^, ^134^, ^214^ (Figure 4B). The correct formation of disulphide bridges within each domain is essential for proper protein folding, which is necessary for eliciting functional transmission blocking antibody responses.^215^ Production of correctly folded full‐length Pfs48/45 with sufficient yield has had highly variable success rates in different heterologous expression systems including baculovirus‐insect cells,^134^ *E. coli*,^216^ Vaccinia virus,^217^ yeast,^218^ *Nicotiana benthamiana*,^219^, ^220^ green algae^221^ and *Drosophila melanogaster* S2 cells.^222^ It has been demonstrated that the C‐terminal fragment containing six cysteines (6C) (Epitope I) is the target of the most potent transmission blocking mAb (85RF45.1).^223^ Therefore, several truncated versions of Pfs48/45 were produced, both in *E coli* and *Lactococcus lactis,* including a two‐domain fragment containing ten cysteines (10C)^224^, ^225^, ^226^ and the 6C fragment.^227^ Alternative approaches, to overcome problems with recombinant protein expression of Pfs48/45 include the use of DNA vaccines, showing promising results in non‐human primates,^228^, ^229^ and expression of Pfs48/45 as a transgene in *P. berghei* parasites.^145^ After decades of limited success, TBV development of both full‐length and a truncated version of Pfs48/45 now show considerable progress^222^, ^225^, ^226^, ^227^, ^230^, ^231^, ^232^, ^233^; both full‐length Pfs48/45 and R0.6C (Pfs48/45‐6C fused to the N‐terminal region of the glutamate rich protein GLURP‐R0) are being prepared for clinical trials in the near future.

The expression of Pfs230 has been even more challenging and has long lagged behind Pfs48/45. Pfs230 is over 300 kDa in size and contains 14 six cysteine motifs, which has hampered the expression of the full‐length protein (Figure 4C). The first Pfs230‐specific mAbs were generated by intraperitoneal injection of isolated *P. falciparum* macrogametes in mice.^48^ The isolated mAbs against Pfs230 lacked the ability to reduce transmission, a finding that nearly eliminated Pfs230 as a TBV candidate. It was subsequently demonstrated that a complement was required for the blocking activity of these mAbs to Pfs230,^234^, ^235^ and that only mAbs of a complement fixing isotype blocked transmission.^236^

The production of six fragments of Pfs230 allowed the identification of an N‐terminal region of Pfs230 (C‐region) that induces functional antibody response.^237^ A subsequent study aimed to further define the region by expression of smaller fragments of this C‐region^238^; None of the truncated fragments was able to induce a functional antibody response, while the anti‐serum did recognize the native protein on gametes. However, Tachibana et al more recently expressed the N‐terminal C‐region and several truncated versions thereof and demonstrated that antibodies induced by the whole C‐region as well as the truncated (C2, C1 and C0) fragments were able to reduce transmission, even in the absence of complement.^239^ They emphasized that the minimal epitope required to induce functional antibodies is the N‐terminal pro‐domain which does not contain cysteines.

Similar to Pfs48/45, many different expression systems have been used to overcome difficulties of expressing native epitopes of Pfs230. These include *E. coli,* 237, 238, 240, 241 a wheat germ cell‐free system,^239^ *Nicotiana benthamiana*,^242^ the baculovirus‐insect cell system^243^, ^244^ and DNA vaccination.^245^ Importantly, all Pfs230 constructs that have been expressed in the recent years are based on the results of the first study demonstrating that region C is the only part that elicits functional antibodies.^237^ Recently, a systematic approach by Tachibana et al aiming to express 27 different protein fragments confirmed that the first cysteine motif is the main region for the induction of functional antibodies. Nevertheless, it cannot be excluded that other regions of Pfs230 are also targets of functional antibodies.

Currently, the most advanced Pfs230 construct in the clinical pipeline is the Pfs230D1M construct expressed in yeast. This construct comprises amino acids 542 to 736 and has been conjugated to EPA.^246^ Sera of rabbits immunized with Pfs230D1M‐EPA in Alhydrogel® were able to block transmission of cultured *P. falciparum* NF54 and two Thai patient isolates in the SMFA. Safety studies have been performed in US and Malian adults with this construct formulated in Alhydrogel® (ClinicalTrial.gov NCT02334462) and AS01 (ClinicalTrial.gov NCT02942277). The results of these trials have to date not been reported. Recruitment is ongoing for a phase 2 trial using the Pfs230D1M‐EPA/AS01 vaccine. In this trial, groups of healthy Malian children of decreasing age will be recruited (ClinicalTrial.Gov NCTO03917654).

While clinical trials are ongoing, more potent Pfs230‐based formulations are being developed, including the conjugation of Pfs230D1M to the outer membrane protein complex which induces higher TRA responses in mice than Pfs230D1M‐EPA.^247^

*Plasmodium vivax* TBV research has moved along the same path as that of *P. falciparum,* although the inability to culture *P. vivax*‐infected RBC in vitro (and thus gametocytes) has limited vaccine discovery and evaluation.

The best characterized *P. vivax* TBV candidate to date is Pvs25.^203^ Early studies demonstrated that Pvs25 has a superior ability to induce potent transmission blocking antibodies compared to another ookinete surface protein Pvs28.^248^, ^249^ Phase I clinical trials in healthy US adults using full‐length Pvs25 expressed in *Saccharomyces cerevisiae* (Pvs25H)^250^ formulated in Alhydrogel® resulted in only modest induction of transmission blocking antibodies.^251^ Preclinical adjuvant optimization studies demonstrated potent induction with Pvs25H formulated in Montanide,^252^, ^253^, ^254^ but a phase I clinical trial was terminated due to unexpected high reactogenicity related to the adjuvant.^204^ This stimulated efforts to develop different vaccine antigen formulations with higher potency and lower undesirable reactivity, such as viral delivery systems^255^, ^256^ or fusion to carrier molecules.^257^, ^258^, ^259^ It remains to be investigated whether these formulations are safe and improve Pvs25 immunogenicity in humans.

Full‐length Pvs48/45 has been recombinantly expressed in *E. coli* with intact native epitopes as demonstrated by the reactivity of antibodies in sera from Colombian individuals.^185^ Potent antibodies were induced in immunization studies using mice and monkeys as demonstrated by complete transmission blocking activity in DMFAs using three natural isolates. Similar to Pfs48/45, Pvs48/45 recombinant protein expression has been challenging; Tachibana et al^260^ used DNA immunizations to overcome problems with protein folding. They immunized rodents with both full‐length *Pvs48/45* and the C‐terminal cysteine rich domain equivalent to Pfs48/45 6C. Antibodies against full‐length Pvs48/45 showed superior reactivity against protein lysates and reduced oocyst numbers. Whether antibodies against Pvs48/45 6C were able to reduce oocyst numbers has not been tested. Pvs47, orthologue of Pvs45/45, has also been tested in this study and antibodies induced after DNA immunization were also able to reduce oocyst number. Using a similar approach, rodent immunizations with a fragment of Pvs230 based on the Pfs230C region (excluding the pro‐domain) also demonstrated the induction of functional antibody responses.^261^ Interestingly, the reduction of oocyst numbers in the DMFA was not dependent on the addition of complement. These preliminary data indicate a potential for these pre‐fertilization antigens as TBV, but more extensive studies will be required to confirm feasibility.

Several TBV candidates (eg Pfs25,^204^, ^211^, ^212^, ^213^ Pfs230 (Clinicaltrials.gov NCT02334462 and NCT029442277) and Pvs25^204^, ^251^) have been tested in clinical studies in the last two decades and Pfs48/45 based vaccine constructs are currently being prepared for clinical testing. In addition, transmission blocking mAbs are increasingly being considered to reduce transmission in exceptional circumstances and support vaccine development.

## 6 TRANSMISSION BLOCKING MONOCLONAL ANTIBODIES

The importance of naturally acquired immunity in malaria was demonstrated in the 1960s by the passive transfer of polyclonal antibodies from adults to infected children, reducing parasitemia and alleviating (severe) clinical disease.^262^ It is increasingly recognized that the B‐cell repertoire of individuals who have been exposed to and are protected against an infectious disease can be a rich source of highly potent mAbs. For human use, mAbs should be potent, target conserved epitopes and preferably be of human origin.

The first human mAbs against a sexual stage antigen were derived from transgenic mice, ie mice expressing human immunoglobulins, that were immunized with Pfs25. Characterization of these mAbs revealed sites associated with transmission blocking activity but also demonstrated that these mAbs have low potency.^263^ More recently, anti‐Pfs25 mAbs were isolated from a human volunteer who was immunized with Pfs25. These mAbs target three different epitopes, two of which had been identified in the transgenic mice study. Interestingly, one of these human mAbs is the most potent anti‐Pfs25 mAb described to date.^264^ A large panel of (potent) rodent mAbs against other sexual stage targets is available and could be of interest for therapeutic use upon humanization.^265^ Many of these mAbs were isolated after immunizations described above that contributed to the identification of P230 and P48/45 and showed high potency in the SMFA^48^, ^107^, ^234^, ^235^ (Table 1).

**Table 1:** Selected transmission blocking monoclonal antibodies

Target	Name	Target epitope	Source	Isotype	Potency in SMFA
Pfs230	63F2A2.2a^236^	Unknown	Mouse	IgG2a	80% at 1 μg/mL, 100% at 4 μg/mL
	P5E2‐2F7‐2B4^234^	Unknown	Mouse	IgG2a	72% at 10 μg/mL, 97% at 30 μg/mL
Pfs48/45	85RF45.5^223^	Epitope V	Rat	IgG2a	79% at 25 μg/mL, 98% at 50 μg/mL,
	32F3^48^, ^223^	Epitope I	Mouse	IgG2b	61% at 12.5 μg/mL, 99% at 25 μg/mL
	85RF45.1^223^, ^267^	Epitope I	Rat	IgG1	IC_80_ = 1‐2 μg/mL
	TB31F^267^	Epitope I	Humanised rat antibody	IgG1	IC_80_ = 0.5‐1 μg/mL
Pfs25	4B7^263^, ^269^	Site 1a/b, EGF3	Mouse		IC_80_ = 29 μg/mL
	32F81^48^, ^311^	LDTSNPVKT peptide on EGF3	Mouse	IgG1	>80% at 10 μg/mL
	AB1245^263^	Site 2, EGF1‐4	Transgenic mouse	Generated as IgG1	IC_80_ = 263 μg/mL
	AB1269^263^	Site 1a/b, EGF3	Transgenic mouse	Generated as IgG1	IC_80_ = 63 μg/mL
	2530^264^	Site 3, mainly EGF2	Human		IC_80_ = 65 μg/mL
	2544^264^	Site 1, EGF1/3/4	Human		IC_80_ = 16 μg/mL
Pf11‐1 (Pfs2400)	mAb1A1^162^	Nonamer repeat [PEE(L/V)VEEV(I/V)]_2_	Mouse	IgG1	70% & 80% at 93 μg/mL, 58% at 44 μg/mL and 57% at 9 μg/mL
Pfs47	IB2^154^	Central region of domain 2	Mouse		66%, 70%, 84% & 88% at 200 μg/mL (4 feeds)
	BM2^154^	Central region of domain 2	Mouse		74% & 94% at 200 μg/mL (2 feeds)

Note

For each target the most potent and human(ized) monoclonal antibodies (mAbs) are given. Note that information about potency is limited for many mAbs since these have often been tested at few, unknown or unspecified concentrations. All mAbs against Pfs230 are complement dependent, unlike mAbs against other targets.^235^, ^236^

Monoclonal antibody 63F2A2.2a is the most potent Pfs230 mAb to date. The most potent Pfs48/45 mAbs described to date target epitope I on the C‐terminal 6‐Cys domain.^215^, ^223^ mAb 85RF45.1 achieves >80% TRA at 1‐5 μg/mL^223^, ^266^, ^267^ and is currently being developed for clinical testing. The variable sequence of the heavy and light chains was used to identify the closest human germline homologues and design a humanized antibody, TB31F, that had a similar affinity and potency as the parental mAb.^267^ Furthermore, crystal structures of both 85RF45.1 and TB31F with the C‐terminal 6‐Cys domain revealed that the antibodies target a highly conserved site on Pfs48/45.^222^, ^267^ This suggests that TB31F will be effective against most *P. falciparum* strains, a finding that can be confirmed using DMFA with genetically diverse strains in field settings. Interestingly, TB31F appears more stable than 85RF45.1, with higher aggregation and melting temperatures,^267^ highlighting one of many possibilities to engineer antibodies to introduce desired characteristics.^268^ Although transmission reducing mAbs against other sexual stage targets such as Pfs25,^48^, ^263^, ^269^ Pf11‐1,^162^ Pfs47^154^ have been identified, mAbs against Pfs230 and Pfs48/45 appear to be the most potent and may therefore be prioritized (Table 1). Rodent mAbs with TRA against *P. vivax* have been described. Crystallization of one of these in complex with Pvs25 revealed its binding site; however, the potency of this mAb has not been established.^270^ Monoclonal antibodies against *P. vivax* target unknown antigens and/or have very limited information on potency.^123^, ^183^, ^271^, ^272^ Better characterization of these mAbs and identification of novel human mAbs will thus be required before considering clinical development of mAbs that target *P. vivax* transmission.

Isolation and characterization of human mAbs can also guide vaccine design, an approach that has been pioneered in the quest for a broadly neutralizing HIV vaccine.^273^ Only recently have researchers started isolating human mAbs against malaria antigens. Two independent groups isolated B‐cells from volunteers who were immunized with sporozoites and identified potent mAbs against Circumsporozoite Surface Protein (CSP) that are unique in binding both the NANP repeat as well as the junctional epitope.^274^, ^275^ Not only are these good candidates for therapeutic antibodies, they also provide valuable information for vaccine design. Strikingly, the CSP targeting vaccine RTS,S (Mosquirix) lacks the junctional epitope. It is tempting to speculate whether including this epitope may increase vaccine efficacy.^274^, ^275^, ^276^ Another example of how mAbs can inform vaccine design comes from the structural studies of 85RF45.1 in complex with Pfs48/45 that describe epitope I.^222^, ^267^ Since antibodies against the epitope I are very potent, vaccine design should aim to direct antibody responses against this conserved epitope. This could for instance be achieved by either reducing immunogenicity of other regions on Pfs48/45 through amino acid mutations and glycosylation or coupling of the antigen to virus‐like particles in such a way that epitope I is well presented to cross‐link B‐cell receptors.

It is anticipated that many human mAbs against sexual stage targets will become available in the near future, either from individuals who are naturally exposed to gametocytes and exhibit high levels of TRA^41^, ^80^ or from individuals taking part in ongoing clinical trials with Pfs230 (ClinicalTrial.gov NCT02334462, NCT02942277, NCTO03917654) or forthcoming trials with Pfs48/45. These developments will plausibly support the development of next generation vaccines and may potentially warrant further clinical development of mAbs for passive immunization.

## 7 EVALUATION OF MALARIA TRANSMISSION BLOCKING VACCINES

### 7.1 Preclinical testing

TBVs and mAbs are recognized as tools with great promise for malaria elimination initiatives. As a consequence of the increased interest in elimination, the pipeline of candidate TBVs and mAbs with transmission blocking properties has expanded considerably in recent years,^178^, ^206^, ^232^, ^233^, ^257^, ^277^, ^278^, ^279^, ^280^ not only in terms of antigens but also in terms of adjuvant formulations and delivery platforms. While TBVs are most likely to be used in combination with vaccines targeting other stages,^281^ guidelines of the US Food and Drug Administration stipulate that vaccine components require efficacy assessments as stand‐alone products. There is currently no consensus on protocol design and endpoints of clinical trials with transmission blocking interventions. Given the complexity and expense of phase III field evaluations that are likely to involve cluster‐randomized trials (CRT), it is, however, evident that robust pipelines for candidate prioritization are needed.^233^ The early evaluation of TBV candidates currently depends on the SMFA where reductions in oocyst density or prevalence are used as indicators of vaccine potency.

Importantly, these in vitro assays are only available for *P. falciparum* and not for *P. vivax* for which parasite culture is currently not possible. In addition, SMFA typically relies on a single gametocyte‐producing *P. falciparum* parasite line at unnaturally high gametocyte densities in combination with a single mosquito strain.^282^ While the value of the SMFA for early TBV evaluation is beyond dispute, the assay has clear limitations when estimating the public health impact of TBVs. The only *P. falciparum* TBV with published results from a field study to date (Pfs25‐EPA as described above) showed excellent results in in vitro assays but failed to induce substantial transmission blocking activity when serum of vaccinated volunteers from the United States was offered to mosquitoes with cultured gametocytes.^213^ The candidate proceeded to a clinical trial in Malian adults where it showed limited efficacy.^212^ This experience illustrates the challenges with early evaluation assays and the urgent need for a model to accelerate vaccine development and identify early failures of vaccine candidates.

One approach that allows preclinical samples, eg serum samples from rodents, in a real‐life context is to add these antibody samples to giRBCs of naturally infected individuals in the DMFA. These natural giRBC gametocyte donors can be infected with multiple gametocyte‐producing clones,^283^, ^284^ thus allowing the testing of antibodies induced in preclinical studies against genetically diverse and complex gametocyte infections at natural gametocyte densities using locally relevant mosquito populations.^282^ This approach has been utilized to assess the efficacy of preclinical and clinical samples for *P. falciparum* TBVs Pfs48/45, Pfs230 and Pfs25^81^, ^178^, ^285^ and for preclinical samples for *P. vivax* TBVs Pvs48/45, Pvs230 and Pvs47 as described above.^185^, ^260^, ^261^ This ex vivo assessment of antibody efficacy may be used to support the interpretation of early immunogenicity trials and explore possible challenges with genetically diverse gametocytes and escape mutants.

## 8 EARLY CLINICAL TESTING

An early evaluation model has recently been developed that may allow a direct assessment of vaccine efficacy against gametocytes that are induced in the vaccinated donor.

Controlled human malaria infection (CHMI) models provide powerful tools for early evaluation of malaria vaccine candidates and have been used predominantly for pre‐erythrocytic^286^, ^287^, ^288^ and blood stage^289^ vaccines. CHMI involve deliberate and controlled infection of malaria naive individuals with either sporozoites (by mosquito bite or intravenous injection of cryopreserved sporozoites) or parasitized erythrocytes (iRBCs).^289^, ^290^, ^291^, ^292^ The appearance and timing of blood stage parasites can be used as an endpoint for CHMI testing pre‐erythrocytic vaccines. Parasite multiplication rate can be used as an endpoint for blood stage^286^, ^289^ vaccines. For TBV, CHMI‐transmission models were recently developed using mosquito bite or iRBC inoculation with *P. falciparum* 3D7 parasites. Treatment of asexual parasites with a subcurative dose of piperaquine monotherapy allows the production of viable mature gametocytes whose infectivity can be assessed by DMFA or direct skin feeding experiments. Both mosquito bite^21^ and iRBC inoculation^33^ result in the production of male and female gametocytes in all volunteers. Gametocyte production is markedly higher following iRBC inoculation, for unknown reasons, and results in a higher likelihood of volunteers infecting mosquitoes by either DMFA or direct skin feeding. This model makes it possible to test the efficacy of highly efficacious TBVs for *P. falciparum* in small groups of 10‐20 malaria naive volunteers per study arm, all infected with a single well‐characterized parasite line (M. Alkema, I. J. Reuling, G. de Jong, K. Lanke, L. E. Coffeng, G‐J. van Gemert, M. van de Vegte‐Bolmer, Q. de Mast, R. van Crevel, K. Ivinson, C. F. Ockenhouse, J. S. McCarthy, R. Sauerwein, K. A. Collins, & T. Bousema, unpublished). A CHMI‐transmission model with similar efficiency in terms of induction of transmissible gametocytes has been developed for *P. vivax* (K. A. Collins, C. Y. T. Wang, M. Adams, H. Mitchell, G. J. Robinson, M. Rampton, S. Elliott, A. Odedra, D. Khoury, E. Ballard, T. B. Shelper, L. Lucantoni, V. M. Avery, S. Chalon, J. J. Moehrle, & J. S. McCarthy, unpublished). At present, CHMI‐transmission studies have only been conducted in malaria naive volunteers, with the advance of CHMI studies in naturally exposed populations,^293^, ^294^, ^295^ it is only a matter of time until CHMI‐transmission studies will also be conducted in endemic settings.

Conventional phase II trials in adult volunteers in endemic settings may complement the clinical development pipeline for TBVs. In addition to immunogenicity and activity of serum samples of vacinnees in the SMFA, such studies can examine the transmissibility of possible naturally acquired infections by DMFA or direct skin feeding.^212^ While incident infections in these studies, unlike CHMI‐trans, are unpredictable and transmission endpoints may be underpowered unless sufficiently high gametocyte densities (eg >5 gametocytes/μL^31^, ^33^ are observed in a considerable fraction of study participants, they allow preliminary efficacy assessments against local parasite strains.

### 8.1 Public health endpoint for TBVs

Definitive evidence on the public health endpoints of TBVs will require larger studies that are complex due to the intended outcome of vaccination that aims to reduce incident infections by reducing the reproductive number (R0) of malaria and overall exposure to infective mosquitoes.^296^ As such, TBV confers a delayed individual benefit^297^ that is achieved by reducing the number of mosquitoes that become infected when biting on vaccinated individuals^296^ and thereby progressively reducing the force of infection over multiple transmission cycles.^298^ The classic approach to phase III clinical trials with TBV, supported by regulatory agencies in consultation with the malaria research community, is to conduct CRT where clusters (eg villages) are randomized to receive either TBV vaccination or no TBV vaccination (control arm).^297^ A cohort of participants in each cluster may be followed for incident infections by molecular or conventional diagnostics as definitive evidence for a reduction in the force of infection.^299^ Infectivity of vaccinated and unvaccinated individuals to mosquitoes and *Plasmodium* sporozoite rates in field‐caught mosquitoes are among the obvious secondary endpoints, and should provide insight into the mechanism of actions of TBV. As surrogate endpoints, it has been debated whether these outcome measures would allow for accelerated approval,^297^ thereby allowing registration and postponing (but not annulling) the requirement to demonstrate the public health benefits of TBV until after vaccine implementation. No consensus has been reached in this discussion. The expectation that the largest effect size of TBV will be observed in low endemic settings (eg EIR < 8 infectious bites/year^300^) or an incidence of infection below 0.2/person/year^301^ and the large heterogeneity in malaria transmission intensity between and within clusters, implies that cluster‐randomized trials will need to be large, involve many clusters and will be costly.

## 9 CONCLUDING REMARKS

Early malaria research was dominated by investigations of the asexual blood stage that is the cause of clinical disease and mortality. Following the release of the first global malaria elimination framework in 2007,^302^, ^303^ attention has shifted to include human‐to‐ mosquito transmission as an important research interest. These efforts have improved our understanding of gametocyte biology as well as the epidemiology and infectivity of gametocytes. *P. falciparum* has been the primary focus of investigations to date, and our understanding of *P. vivax* gametocyte biology is limited. Several independent studies reported the presence of gametocyte antigens on the *P. falciparum* giRBC membrane surface. While data are in part conflicting^77^, ^78^, ^79^, ^80^ giRBC immunity may contribute to the large variation in gametocyte production observed in natural infections. Whether *P. vivax* giRBC immunity exists remains unknown.

While the existence of TRA is beyond dispute, and several key antigens are firmly established, the importance of TRA in determining natural transmission dynamics remains to be quantified.^81^, ^171^ This is particularly true for *P. vivax* where very few studies have examined the occurrence of gametocyte immunity and TRA in endemic populations or whether the kinetics of TRA differs with respect to mosquito‐derived infection versus relapsing infection.

Compared to TRA, the potential importance of TE for the transmissibility of natural infections is very poorly understood. Understanding the dynamics of TRA and TE following natural exposure and following vaccination may be particularly important for malaria elimination initiatives. Some evidence suggests that malaria transmission efficiency may increase as the burden of malaria decreases^304^; it is conceivable that waning gametocyte immunity may play a relevant role in this. Future research should quantify the acquisition and waning of TRA in relation to malaria exposure, prove or disprove the existence of TE and unravel the associated immune profiles that may include other targets than the well‐established Pfs48/45 and Pfs230.^41^, ^81^, ^108^, ^110^, ^114^

TBVs may play important roles in the malaria endgame, preventing secondary infections from the remaining pockets of transmission, preventing malaria outbreaks and protecting other interventions from escape mutants.^281^ The discovery of giRBC immunity opens new avenues for TBV development. Great progress has also been achieved with conventional TBVs. Pfs25 has been the first vaccine to be tested in naturally exposed individuals,^212^ providing invaluable insights for future field trials, and great progress has been achieved with TBVs targeting prefertilization antigens. Several prefertilization TBV candidates are currently in, or approaching, clinical testing. Next generation TBVs may include novel immunogens or a combination of known antigens. The future inclusion of multiple antigens may decrease the proportion of vaccinees who respond poorly,^305^ reduce the risk of escape mutant and potentially result in a synergistic effect.^277^, ^306^, ^307^, ^308^, ^309^ Currently, the most logical dual‐antigen vaccine would include the top candidates Pfs48/45 and Pfs230 and preliminary data of a chimeric protein showed potential synergy.^310^ The increased pipeline of vaccine candidates and mAbs that aim to reduce transmission is promising and generates a sense of urgency to reach consensus on study designs to prioritize candidates and accelerate testing and implementation processes of lead candidates. The expectation that cluster‐randomized trials for TBVs will be large and costly makes it unlikely that the international community can afford such trials for many TBV candidates. This is a strong argument to invest in early evaluation models, improve our understanding of transmission and continue to investigate naturally acquired and vaccine‐induced immunity against gametocyte antigens.
